# Unveiling Microbial Diversity: Raman Spectroscopy’s Discrimination of *Clostridium* and Related Genera

**DOI:** 10.1021/acs.analchem.4c03280

**Published:** 2024-09-18

**Authors:** Markus Salbreiter, Annette Wagenhaus, Petra Rösch, Jürgen Popp

**Affiliations:** † Institute of Physical Chemistry, Friedrich Schiller University Jena, Helmholtzweg 4, Jena D-07743, Germany; ‡ Leibniz Institute of Photonic Technology Jena - Member of the Research Alliance, Leibniz Health Technologies, Albert-Einstein-Str. 9, Jena D-07745, Germany; § InfectoGnostics Research Campus Jena, Philosophenweg 7, Jena D-07743, Germany

## Abstract

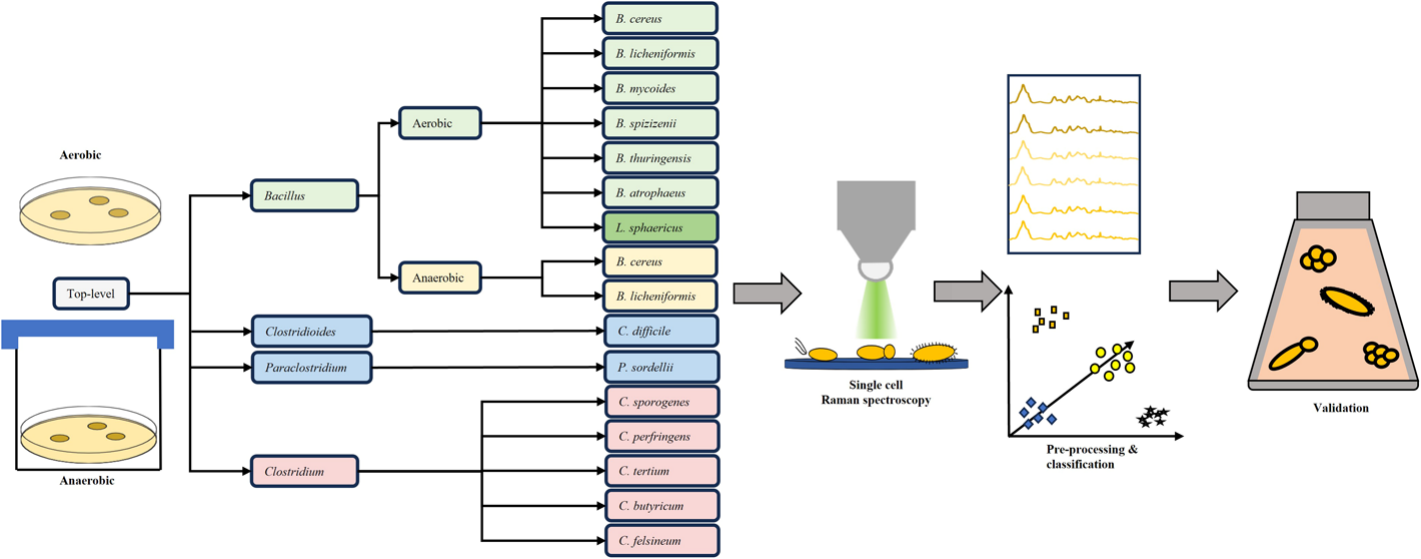

In the clinical environment, the identification of phylogenetic closely related genera and species like *Clostridium* and *Bacillus spp.* is challenging. Both genera contain representatives of pathogenic and nonpathogenic species that need to be distinguished for a proper diagnostic read-out. Therefore, reliable and accurate detection methods must be employed for the correct identification of these genera and species. Despite their high pathogenicity, clostridial infections and food contaminations present significant challenges due to their unique cultivation conditions and developmental needs. Therefore, in many diagnostic protocols, the toxins are used for microbiological documentation. However, the applied laboratory methods suffer in accuracy, sometimes require large bacterial loads to provide reliable results, and cannot differentiate pathogenic from nonpathogenic strains. Here, Raman spectroscopy was employed to create an extensive Raman database consisting of pathogenic and nonpathogenic *Bacillus* and *Clostridium* species. These genera, as well as representatives of *Paraclostridium* and *Clostridioides* were specifically selected for their phylogenetic relation, cultivation conditions, and metabolic activity. A chemometric evaluation of the Raman spectra of single vegetative cells revealed a high discriminating power at the genus level. However, bacilli are considerably easier to classify at the species level than clostridia. The discrimination between the genera and species was based on their phylogeny and not their aerobic and anaerobic cultivation conditions. These encouraging results demonstrated that Raman spectroscopy coupled with chemometrics is a robust and helpful method for differentiating *Clostridium* species from *Bacillus*, *Clostridioides*, and *Paraclostridium* species. This approach has the potential to be a valuable tool in clinical diagnostics.

## Introduction

*Clostridium* is a diverse genus of Gram-positive, anaerobic, endospore-forming, rod-shaped bacteria. It includes historically infamous pathogens such as *Clostridium botulinum* and *Clostridium tetani* as well as benign strains used in industrial fermentations, most notably *Clostridium acetobutylicum* and *Clostridium beijerinckii*.^1^ Since its description in 1880 by Prazmowski,^2^ the genus has included numerous bacterial species, with up to 200 species currently recognized worldwide. The genus’s prominence is reflected in the more than 54,500 entries in the PubMed database^3^ and the approximately 6,600 genomic sequences submitted in the GenBank database.^4^ The majority of these harmless nonpathogenic bacteria are found in the environment, on plants, on the skin, and in the mucosa of animals and humans, particularly in the intestinal tract. They are able to produce endospores, which enables them to withstand harsh environments. Furthermore, they lack a respiratory chain (e.g., cytochrome), thus they obtain ATP by substrate-level phosphorylation. It is therefore evident that many species within the genus can therefore be distinguished by their anaerobic energy-yielding mechanisms, which may be proteolytic or saccharolytic.^5^

Diagnosis of clostridia infections and food contamination is challenging due to their distinctive developmental requirements. A total of 40 to 50 species have been linked to clinical illnesses in domestic animals and humans. Of these, 30 are mild infections and 15 are serious pathogens.^6,7^ Toxins produced by major pathogenic species result in the formation of lesions and the development of clinical symptoms. These toxins are among the most potent in the microbial world and continue to serve as a basis for the identification of several major pathogenic species.^8−10^ In recent years, there has been a surge in interest in clostridial strains for various reasons. The recent revival of interest in the use of clostridial fermentations for biofuel production is evidenced following sources.^11^ The agricultural sector has recently demonstrated a renewed interest in necrotic enteritis and gas gangrene caused by *Clostridium perfringens*, particularly in light of regulatory changes that have reduced the use of antibiotic supplementation in animal feed.^12,13^ Medical interest has increased in the newly designated “superbug” *Clostridioides difficile* (formerly known as *Clostridium difficile*).^14,15^ Furthermore, *Paraclostridium sordellii* (formerly known as *Clostridium sordellii*)^16^ is of significant medical interest due to its capacity to cause pneumonia, endocarditis, arthritis, peritonitis, and myonecrosis.^17^ In the medical field, the development of rapid and accurate detection methods for pathogenic *Clostridium* species is of vital importance. Novel approaches must be explored and developed to address this need.

One such approach is Raman spectroscopy (RS). In this approach, the target’s vibrational information is acquired as spectral information, which subsequently allows the characterization and identification of the microbe. In comparison to other identification methods, Raman spectroscopy is relatively inexpensive, rapid, and straightforward to perform, while also being nondestructive, culture- and label-free.^18^ Raman spectroscopy captures the entire biochemical composition of a sample (“spectroscopic molecular fingerprint”), which can be characterized and differentiated using chemometric methods.^19,20^ This enables the characterization, distinction, and identification of bacteria at the species and even subspecies level.^21^ In a clinical setting, this would be invaluable, as it would facilitate the identification of the causative pathogen and a rapid diagnosis.

Although *Clostridium* species are of significant medical importance, only a limited number of Raman spectroscopy studies have been conducted with *Clostridium* species on a large scale. To the best of our knowledge, Schuster et al. were the first group to implement Raman spectroscopy for the characterization of two separate clostridial species in two different studies. The initial study focused on the Raman analysis of individual *Clostridium acetobutylicum* cells. The results demonstrated that cell differentiation could be discerned, thereby enabling the investigation of heterogeneity and the identification of subpopulations with diverse cell compositions on a single-cell level.^22^ The second study proceeded to investigate the chemical composition of single-cell analysis of *Clostridium beijerinckii* cells in a greater depth. It was able to detect all major cell components, including the storage polymer granulose.^23^ Subsequently, Zu and colleagues conducted two further studies involving clostridial species. The initial study employed predictive modeling and multivariate data analysis in *C. acetobutylicum* fermentations for real-time culture monitoring. Real-time Raman data was obtained from cultures of *C. acetobutylicum* grown on glucose for comparative off-line product analysis. Subsequently, partial least-squares (PLS) models were constructed for both agitated and static cultures, with the objective of monitoring the media components and metabolites present (e.g., glucose, butyric acid, acetic acid, and butanol). Agitation experiments were conducive to modeling, while static experiments were characterized by greater chaos, particularly during and after manual sampling.^24^ The second study by Zu and colleagues employed Raman-aided metabolomics to track metabolites in glucose-fed *C. acetobutylicum*, which built upon their previous experiments and results. PLS models were constructed using data obtained from *in situ* Raman spectroscopy probes and high-performance liquid chromatography (HPLC) analysis. The predictions of the PLS demonstrated a high degree of correlation with the measured data, thereby establishing their potential as valuable tools for the characterization, process control, and optimization of cultures.^25^

Raman spectroscopy was employed to identify *Clostridioides difficile* infections (CDI) in serum samples produced by the impact of their toxins A and B. Serum samples were either spiked with toxin A, B, or both and then analyzed using Raman spectroscopy. The PLS-LDA and SVM models demonstrated 100% accuracy in distinguishing toxin-spiked sera from control serum, with sensitivity varying from 87 to 100%, while specificity ranged from 77 to 100% depending on concentration.^26^ Koya et al. also analyzed *C. difficile* toxin-spiked stool samples with varying TcdA and TcdB concentrations and various chemometric models.^27^ The accuracy of the models ranged from 64 to 77% for all models, with sensitivity and specificity varying from 69 to 90% and 43 to 78%, respectively.^27^ Zhang et al. employed confocal Raman microspectroscopy (CRM) in conjunction with chemometrics to detect and to identify *C. botulinum* on a species and serotype level.^28^ This data set included *C. difficle*, *C. perfringens*, *C. botulinum*, and two botulism-causing strains, *C. botulinum* type A and type B. A PCA model was used to distinguish between the three species, and a PCA-linear discriminant analysis (LDA) model was utilized to distinguish between the serotypes. The study demonstrated that CRM paired with chemometrics can be used to distinguish *C. botulinum* serotypes.^28^ Furthermore, the application of surface-enhanced Raman spectroscopy (SERS) in conjunction with chemometrics enabled the rapid and accurate analysis of individual food-borne pathogens including *C. difficile* and *C. perfringens*.^29^ Additionally, SERS was also employed to rapidly distinguish between individual spore-forming bacteria (e.g., *C. perfringens* and *Bacillus spp.*) in spice powders.^30^

In this study, *Bacillus* and *Clostridium* as well as representatives from *Paraclostridium* and *Clostridioides* were specifically selected for their phylogenetic relation and their ability to produce endospores. Furthermore, these genera are capable of growing in either aerobic or anaerobic environments. Raman spectroscopy was employed to generate a comprehensive database of Raman spectra of single vegetative cells belonging to various species, including both pathogenic and nonpathogenic organisms. Subsequently, a data set was compiled, and chemometric analysis was conducted. Furthermore, the classifier was validated by means of an independent validation process, which involved the use of additional spectra.

## Materials and Methods

### Strains and Cultivation Conditions

A comprehensive overview of the *Bacillus* and *Clostridium* species and strains used throughout this study is provided in Table S1. Most of the pathogenic and nonpathogenic strains of *Bacillus* and *Clostridium* were either obtained from the German Collection of Microorganisms and Cell Culture GmbH (DSMZ), Braunschweig, Germany, the American Type Culture Collection (ATCC), Manassas, Virginia, USA, or the Universitätsklinikum Jena, Germany.

All *Bacillus*, *Clostridium*, *Clostridioides* and *Paraclostridium* strains were grown on nutrient agar Petri dishes (NA, peptone from meat 5.0 g/L, yeast extract 2.0 g/L, meat extract 1.0 g/L, agar 15 g/L, pH 7.0) with supplementary 100 mg/L calcium chloride (CaCl_2_x2H_2_O). Some strains, such as *C. difficile* DSM 12056, had to be precultured on Columbia sheep blood agar (VWR, Darmstadt, Germany) before being able to grow on NA. All the chemicals used for the media production were bought through VWR (Darmstadt, Germany) and Merck (Darmstadt, Germany). The aerobic strains were grown at 30 °C for 1 day in a standard incubator. The anaerobic strains were incubated in an anaerobic incubation system by Anaerocult and using Anaerocult A pads for the generation of an anaerobic environment in an anaerobic jar (VWR, Darmstadt, Germany) at 30 or 37 °C for five to 7 days. In addition, the cytochrome c of the horse heart reference sample was obtained at Merck (Darmstadt, Germany) as a red powder.

### Sample Preparation

Using sterile 1 mL dH_2_O, the cultivated strains were scraped from the plate and transferred into Eppendorf tubes (VWR, Darmstadt, Germany). The cell suspension was then washed and centrifuged three times with sterile 1 mL dH_2_O at 5000 rpm for 5 min at 4 °C. Afterward, the pellet was resuspended in 200 μL dH_2_O. Finally, 10 μL of the suspension was taken up with an Eppendorf pipet and tiny droplets were placed on the nickel foil. Serial dilutions were performed if the suspension was too turbid, or the cell count was too high for single cell measurements. Raman measurements were only performed on single, morphologically intact cells that were clearly isolated from other cells. The cytochrome c reference sample was prepared by adding very little amounts onto the Ni-foil and spreading it out evenly.

### Raman Spectroscopic Instrumentation and Data Analysis

All Raman spectroscopic measurements were performed by using the Raman microscope BioParticleExplorer (rap.ID Particle Systems GmbH, Berlin, Germany). The light was produced by a solid-state diode-pumped frequency-doubled Nd:YAG laser module with an excitation wavelength of 532 nm (LCM-S-11-NNP25, Laser-export Co. Ltd., Moscow, Russia). An Olympus MPL-LFN-BD 100x objective (Olympus Corporation, Tokyo, Japan) was used to focus the laser light onto the sample with a spot size of less than 1 μm and a maximum laser intensity of about 10 mW. Before being detected using a thermoelectrically cooled CCD (DV 401 BV; Andor Technology), the backscattered Raman light is diffracted by a single-stage monochromator with a 920 lines/mm grating (HE532, Horiba Jobin Yvon, Munich; Germany). As a result, a spectral resolution of roughly 8 cm^–1^ is offered.

Before any bacterial cells could be measured, silicon and 4-acetamidophenol (4-AAP) were subjected to measurements. The silicon was measured at 100% (10 mW) laser power for 0.5 s, while the 4-AAP was measured at 50% (5 mW) for 5 s. Afterward, the bacteria were irradiated with a laser power of 25% (2.5 mW) for 5 s. Spectra of each bacterial strain were gathered in three biological replicates with each containing 100 spectra. Additionally, the cytochrome c spectra were obtained with a laser power of 5% and an exposure time of 15 s.

One of the most important steps of Raman data analysis is spectral preprocessing since it removes unwanted signals and variation within the different Raman spectra, while also enhancing the differences between the different bacterial species spectra. The data preprocessing was performed using the Ramanmetrix software for Raman data analysis containing a vast array of different steps.^31^ The preprocessing of the data contains multiple steps with the first step being the removal of unwanted signals such as cosmic spikes and artifacts by cutting off below 350 cm^–1^ and above 3150 cm^–1^.^32^ Next, the wavenumber calibration on the wavenumber-axis was performed with the spectra of 4-AAP to correct peak position.^33^ A sensitive nonlinear iterative peak (SNIP) clipping algorithm was used to make a baseline correction to remove the fluorescent background.^34^ Finally, the silent region from 1800 to 2600 cm^–1^ was cropped and vector normalization was applied before the preprocessed mean spectra were calculated.

In order to compare the mean spectra of the aerobic and anaerobic bacteria, a combination of principal component analysis (PCA) and support vector machine (SVM) with radial basis kernel was chosen as the most appropriate methodology for the task at hand. This was based on the findings of Chang and Lin,^35^ which demonstrated the efficacy of this approach.^35^ SVMs have been demonstrated to be highly effective models for classifying and identifying different bacterial species, as evidenced by previous studies.^36,37^ The model was subsequently employed for the validation of the independent test data.

### Phylogenetic Analysis

Maximum likelihood 16S rRNA gene sequence-based phylogeny was calculated for the species of *Clostridium*, *Bacillus*, *Clostridioides*, and *Paraclostridium*. The 16S rRNA gene sequences were obtained from the European Nucleotide Archive (ENA)^38^ and the National Center for Biotechnology Information (NCBI)^39^ database for the type strains of the following species: *C. butyricum* (ENA acc. no. M59085), *C. tertium* (ENA acc. no. AJ245413), *C. perfringens* (ENA acc. no. M59103), *C. sporogenes* (ENA acc. no. M59115), *C. felsineum* (ENA acc. no. S46736), *C. difficile* (ENA acc. no. X73450), *P. sordellii* (ENA acc. no. M59105), *B. mycoides* (ENA acc. no. AB021192), *B. thuringiensis* (ENA acc. no. AF290545), *B. cereus* (ENA acc. no. DQ207729), *B. atrophaeus* (ENA acc. no. X60607), *B. spizizenii* (ENA acc. no. AF074970), *B. licheniformis* (ENA acc. no. X60623), and *L. sphaericus* (ENA acc. no. X60639). The obtained sequences were then aligned with ClustalW.^40^ A maximum likelihood phylogenetic tree was calculated from the same alignment with FastTree2.1^41^ employing the GTR+CAT model and 1000 bootstraps replications. The outgroups consist of three 16S rRNA genes from members of the phylum *Actinomycetota*, namely, *Corynebacterium glutamicum* (ENA acc. no. X80629), *Streptomyces albus* (ENA acc. no. AJ621602), and *Mycobacterium tuberculosis* (NCBI acc. no. NR_1028102.2). The calculated 16S rRNA gene sequence-based phylogenetic tree was visualized with iTOL v6 (https://itol.embl.de)^42^ and visually enhanced with Adobe Illustrator.

## Results and Discussion

In this study, the composition of the bacteria was chosen based on their growth conditions, phylogenetic relatedness, clinical relevance, and their ability to produce endospores. The spectra of 7 *Bacillus* and 5 *Clostridium* species as well as 1 *P. sordellii* and 12 *C. difficile* strains were acquired. As previously stated, the Bacilli were grown aerobically, whereas the Clostridia were cultivated anaerobically. In addition, the ability of *Bacillus cereus* and *Bacillus licheniformis* to also grow anaerobically was exploited for the data set.

### Phylogenetic Analysis

**Scheme 1:**
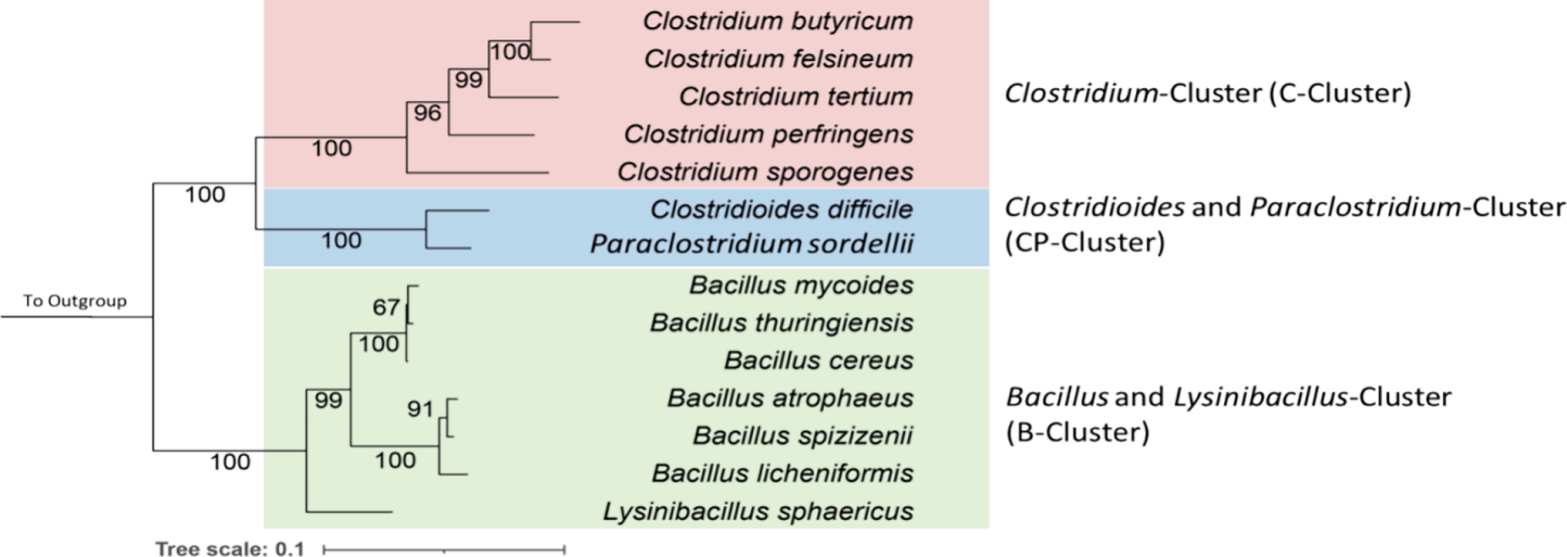
Maximum Likelihood 16S rRNA Gene Sequence-Based Phylogenetic Tree Depicting the Positions of the *Clostridium*, *Clostridioides*, *Paraclostridium*, and *Bacillus* Species Phylogeny was calculated as described in the material and methods section. Bootstrap values after 1000 re-samplings are given at the nodes (in %). The tree scale (branch length values) represents the mean expected rates of substitution per site. The outgroups consist of three 16S rRNA genes from Gram-positive bacteria *Corynebacterium glutamicum*, *Streptomyces albus*, and *Mycobacterium tuberculosis*.

In the phylogenetic tree obtained of 16S rRNA genes, the *Clostridium* and *Bacillus* species as well as representative species and strains of the *Clostridioides* and *Paraclostridium* genera each form their own cluster (Scheme 1). As expected, the *Clostridium* species have formed their own cluster which is well separated from the other genera. *Clostridioides difficile* and *Paraclostridium sordellii* are clearly distinguished from their former genus since they are no longer officially classified as *Clostridium*.^15,16^*Bacillus*, on the other hand, create their own cluster with smaller clusters developing inside it, such as the *Bacillus cereus* group, which contains *B. cereus*, *B. thuringiensis*, and *B. mycoides*, and the *Bacillus subtilis* group, which includes *B. atrophaeus*, *B. spizizenii*, and *B. licheniformis*. *Lysinibacillus sphaericus* (formerly known as *Bacillus sphaericus*)^43^ is distinct from the other *Bacillus* species, but for convenience, we classified it with the *Bacillus* cluster because it was previously included. Since this study was not solely focused on phylogenetic relatedness, three clusters were formed for convenience: *Clostridium*-Cluster (C-Cluster), *Bacillus*-*Lysinibacillus*-Cluster (B- Cluster), and *Clostridioides-Paraclostridium*-Cluster (CP- Cluster).

### Raman Band Assignment

**Figure 1:**
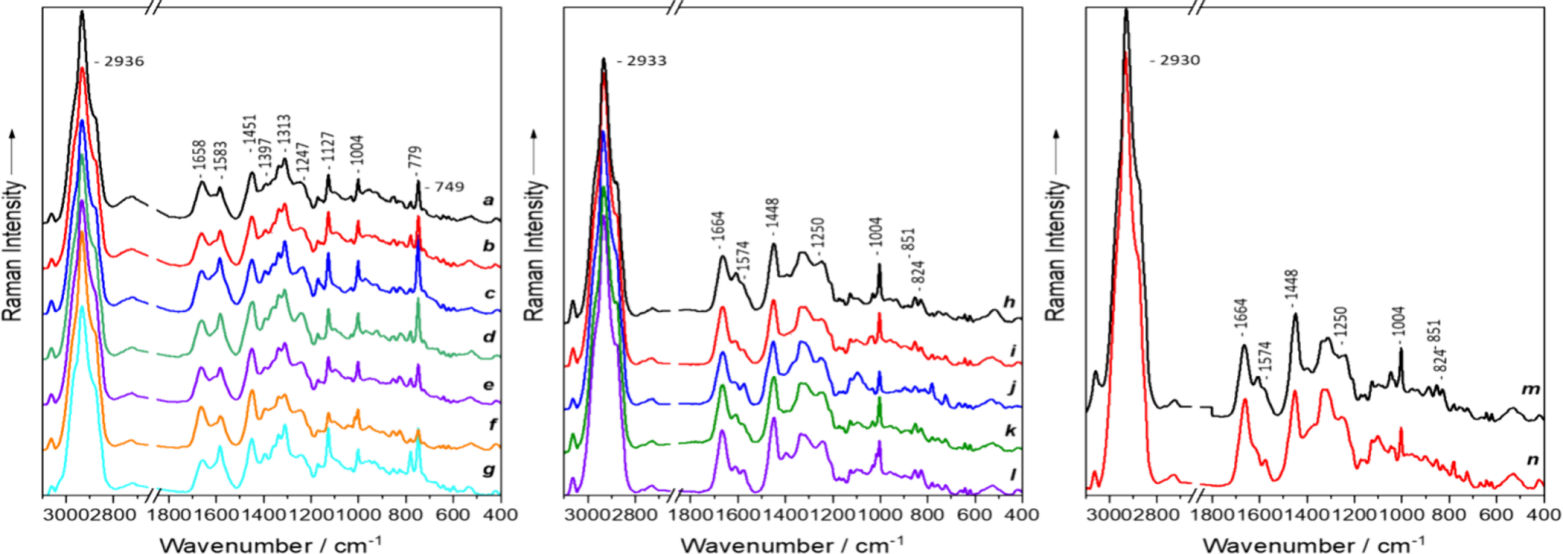
Raman mean spectra of vegetative cells of *Bacillus* (B), *Clostridium* (C), *Paraclostridium* and *Clostridioides* (CP) species: (a) *B. thuringensis*, (b) *B. spizizenii*, (c) *L. sphaericus*, (d) *B. mycoides*, (e) *B. licheniformis*, (f) *B. cereus*, (g) *B. atrophaeus*, (h) *C. tertium*, (i) *C. sporogenes*, (j) *C. perfringens*, (k) *C. felsineum*, (l) *C. butyricum*, (m) *C. difficile*, and (n) *P. sordellii*.

The mean spectra acquired from single *Bacillus*, *Clostridium*, *Clostridioides*, and *Paraclostridium* cells are presented in Figure 1. The bacteria have a well-established spectral pattern, corresponding with usual bacteria spectra, revealing the full range of biomolecules.^18,44,45^ The Raman spectra of a clostridial cell (Figure 1, spectra h–l) consist of the usual spectral signals: The most prominent band featured in the spectra is centered at 2933 cm^–1^^46^ represents the C–H stretching vibrations, while the band at 1448 cm^–1^^47−49^ represents CH_2_/CH_3_ deformation vibrations mainly of lipids and proteins. In addition, the Raman bands at 1664 cm^–1^^50,51^ and 1250 cm^–1^^52^ can be assigned to amide I and amide III vibrations. The main signal contribution at 1664 and 1250 cm^–1^ can be assigned to protein vibrations; however, lipid and polysaccharide signals can also be found here. Furthermore, the bands at 1004 cm^–1^^48^ and 851 cm^–1^^52,53^ are represented by the ring breathing vibrations of phenylalanine and tyrosine, respectively. Lastly, the band at 1574 cm^–1^^50,53^ can be assigned to nucleic acids whereas the second ring breathing mode of tyrosine can be observed at 824 cm^–1^.^54^ In addition, the Bacilli (B) produce specific bands at 1583, 1397, 1313, 1127, and 749 cm^–1^^55,56^ that can be assigned to cytochrome vibrations.^57−62^ The band assignment of the mean Raman spectra of *P. sordellii* and *C. difficile*(CP) is identical to that of the *Clostridium* species. Table S2 in the supplement section provides a more detailed description of the band assignments of all four genera.

### Distribution of Cytochromes

**Figure 2:**
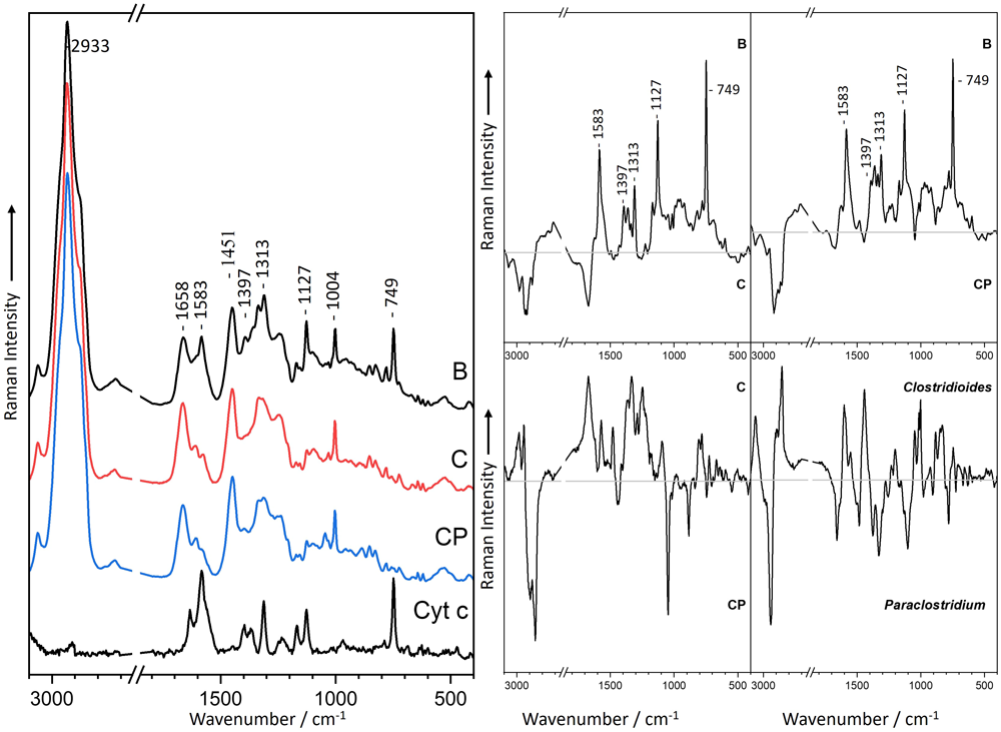
Raman mean spectra of vegetative cells of *Bacillus*-Cluster (B), *Clostridium*-Cluster (C) and *Clostridioides*-*Paraclostridium*-Cluster (CP) as well as cytochrome c (Cyt c) from horse heart as reference (left). Difference spectra were calculated by subtracting the mean spectra (*Bacillus*-*Clostridium*, *Bacillus*-*Clostridioides*, *Clostridium-*CP and *Clostridioides-Paraclostridium*) (right).

To further visualize the occurrence or absence of cytochrome between the three bacterial clusters, a reference sample of cytochrome c was measured, and the Raman bands were then compared to those of the bacterial species. Figure 2 displays a comparison of mean Raman spectra between the three clusters and a pure cytochrome c reference sample from the horse heart. Out of the multiple cytochrome bands found in the reference spectrum (Figure 2), the most prominent bands found in cytochrome were 1583, 1397, 1313, 1127, and 749 cm^–1^,^55,56^ which correspond to the bands found in *Bacillus* but not in *Clostridium* or CP-cluster. To show the differences between the three clusters (B, C, and CP), difference spectra were calculated by subtracting the preprocessed spectra of C or CP from B. The difference spectra of B–C and B-CP in Figure 2 reveal bands at 1583, 1397, 1313, 1127, and 749 cm^–1^,^55,56^ indicating the same distinctive Raman signals as the cytochrome reference spectrum in Figure 2. This underlines the fact that cytochrome was indeed found only in aerobic growth and only in *Bacillus* species.

Cytochromes are heme-proteins in which the heme prosthetic group undergoes oxidation or reduction as part of the protein’s activity. Cytochrome is made up of an iron ion that is coupled to a porphyrin cycle, which includes a conjugated π system of four pyrrole rings linked by vinyl groups.^63^ Porphyrin-containing compounds such as cytochromes exhibit an intense absorption peak (Soret peak) at around 400 nm as well as additional absorption bands at 500 and 560 nm, which means that the electronic absorption of cytochrome is in resonance with the Raman excitation wavelength of 532 nm.^64,65^ The in-plane C=C stretching vibration of the porphyrin skeleton of cytochromes is observed at 1583 cm^–1^ in the Raman spectra of Figure 2.^56,58^ The Raman signal at 1313 cm^–1^ corresponds to the cytochrome ring modes that are coupled with the peripheral vinyl groups.^56,58^ The bands at 1127 and 749 cm^–1^ are due to the deformation modes of CCC and CNC bonds of the chromophore.^56−58^

It is also important to note that resonance Raman active vibrations of heme compounds such as cytochrome can be grouped into heme-molecule-specific symmetries (A_1g_, A_2g_, B_1g_, and B_2g_). Interestingly, the vibration at 1397 cm^–1^ may be attributed to an A_2g_ symmetry, which refers to the antisymmetric vibrations in regard to the C_2_ axis of each pyrrole ring, as well as the fact that the neighboring pyrrole rings vibrate in phase.^64,66^

### Aerbic vs Anerobic Growth

**Figure 3:**
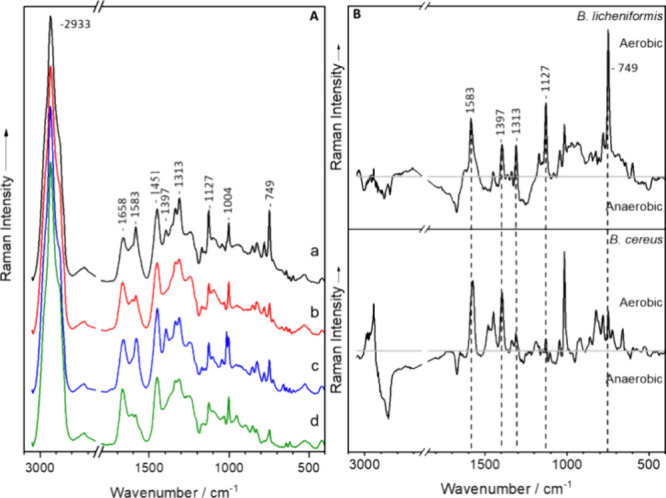
(A) Mean spectra of vegetative cells of *Bacillus licheniformis* (a, b) and *Bacillus cereus* (c, d) grown either aerobically (a, c) or anaerobically (b, d). (B) Difference spectra calculated with mean spectra of *B. licheniformis* (a, b) and *B. cereus* (c, d).

To further underline the fact that aerobic and anaerobic growth can be differentiated using Raman spectroscopy, the ability of *Bacillus cereus* and *Bacillus licheniformis* to grow under both conditions was exploited. *B. cereus* and *B. licheniformis* are well-known facultative anaerobes that can grow anaerobically in the absence of oxygen *via* at least two distinct metabolic pathways: anaerobic respiration using nitrate, glucose, glycerol, or fumarate among others as an electron acceptor and fermentation in the absence of electron acceptors.^67−69^ As previously seen in Figures 1 and 2, the same characteristics of aerobic and anaerobic growth can be visualized in the Raman spectra of these two species (Figure 3). All the typical bacterial Raman signals are shown in their spectra and are comparable with those of the aerobic *Bacillus* and anaerobic *Clostridium* signals. It is also worth noting that the cytochrome bands can only be observed in aerobic cultures (Figure 3a,c) and not in anaerobic cultures (Figure 3b,d).

As already mentioned, the Raman spectra of the *Bacillus* species revealed marker bands specific for cytochrome, which were absent in the *Clostridium* spectra (Figure 1). The specific marker bands for cytochrome can be found at 1583, 1397, 1313, 1127, and 749 cm^–1^.^55,56^ Furthermore, these bands were exclusively visible in aerobic *Bacillus* cultures as opposed to anaerobically cultivated *Bacillus* (Figure 3).

Additionally, the difference spectra in Figure 3 were calculated by subtracting the preprocessed spectra of the aerobically and anaerobically cultivated *B. licheniformis* and *B. cereus*. Both difference spectra exhibit the distinctive cytochrome bands at 1583, 1397, 1313, 1127, and 749 cm^–1^,^55,56^ thus underlining the fact that cytochrome bands were exclusively visible in aerobic culture as opposed to anaerobic cultures.^70,71^

Through this insight, it can be shown that cytochrome is indeed needed for aerobic respiration, whereas it is not needed for anaerobic respiration or fermentation and is in these cases therefore absent from the spectra. *B. cereus* possesses three terminal oxidases in its aerobic respiratory chain, which include cytochromes aa_3_, caa_3_, and bd,^72^ whereas *B. licheniformis* includes multiple c-type cytochromes.^73^ Cytochrome aa_3_ (menaquinol oxidase) has been shown to be a major contributor to respiration in most aerobic growth conditions found in *Bacillus*, whereas caa_3_ (cytochrome c oxidase) and bd (menaquinol oxidase) have more minor roles.^74,75^ Because the resonance Raman spectra of heme compounds are caused by heme vibrations rather than protein vibrations, heme compounds with identical hemes generally have similar Raman spectra.^64^ Therefore, it is difficult to distinguish between cytochrome aa_3_ and caa_3_ since both possess heme A, while cytochrome bd contains heme B and D.^60,61,74^

### Classification, Validation, and Identification

A more comprehensive evaluation of the Raman spectra was carried out by implementing chemometrics. A principal component analysis (PCA) coupled with a support vector machine (SVM) was applied to determine these differences. PCA is a technique used to reduce the dimensionality of big data sets by dividing a large set of variables into a smaller set that retains most of the information. SVM, on the other hand, is a supervised learning approach for classification that is used to generate the optimal margin or hyperplane in an n-dimensional space (n = number of features) that clearly classifies the data points.^35^ Depending on what was being classified, the number of features had to be adjusted.

Initially, the B-, C-, and CP-cluster classifications were used to distinguish between the three clusters, with an accuracy of 82.5% and variable degrees of sensitivity and specificity (Table 1). *Paraclostridium* and *Clostridioides* are intrinsically distinct genera, however they were grouped together to generate a non-*Clostridium* equivalent, which most likely influenced the model’s overall performance. A genus-level classification was undertaken to see whether separating both genera into their own genus would make a substantial change in classification. The classification of the genera was performed to differentiate between *Bacillus*, *Clostridioides*, *Paraclostridium*, and *Clostridium* species. Thus, 4200 spectra from seven distinct *Bacillus* species and 1800 spectra from five different *Clostridium* species as well as one strain of *P. sordellii* and 12 strains of *C. difficile* were measured. Hence, the genus-level SVM classified all 8775 Raman spectra with an accuracy of 84.9% as well as achieving sensitivities and specificities between 72.4 to 99.5%.

**Table 1:** Summary of the Classification Steps at the Cluster, Genus, and Species

**level**		**number of spectra**	**accuracy/%**	**specificity/%**	**sensitivity/%**
cluster	B	4200	82.5	93.2	92.2
	C	1800		90.8	70.7
	CP	2775		90.3	75.4
genus	*Bacillus*	4200	84.9	94.0	92.4
	*Clostridium*	1800		93.0	72.5
	*Clostridioides*	2469		91.8	80.1
	*Paraclostridium*	306		99.5	94.8
species	*Bacillus*(mean)	4200	94.7	99.3	94.9
	*Bacillus atrophaeus*	300		99.0	95.0
	*Bacillus cereus*	1200		97.8	93.2
	*Bacillus licheniformis*	1200		99.4	94.9
	*Bacillus mycoides*	300		98.9	92.7
	*Lysinibacillus sphaericus*	600		99.7	97.7
	*Bacillus spizizenii*	300		99.5	94.0
	*Bacillus thuringensis*	300		99.3	96.7
	*Clostridium* (mean)	1800	91.9	97.8	90.0
	*Clostridium felsineum*	600		96.9	80.7
	*Clostridium butyricum*	600		97.6	88.3
	*Clostridium sporogenes*	600		99.7	96.7
	*Clostridium perfringens*	600		97.8	97.8
	*Clostridium tertium*	600		97.9	90.0

Subsequently, 94.7% of the 4200 *Bacillus* spectra were correctly classified on the species level, while the 1800 *Clostridium* spectra were assigned with an accuracy of 91.9%. There were several misclassifications in the *Bacillus* species discrimination between *B. cereus*, *B. mycoides*, and *B. thuringiensis* since all three species are members of the *B. cereus* group and hence extremely closely related. *B. atrophaeus*, *B. licheniformis*, and *B. spizizenii*, on the other hand, had less misclassifications among themselves, which made sense given that those three species are part of the *B. subtilis* group. Furthermore, there were a few misclassifications between *B. licheniformis* and *B. cereus*, which might be attributed to the inclusion of aerobic and anaerobic cultured strains, making the model more complicated and diverse. Overall, the sensitivity and specificity of the classification ranged from 92.7 to 97.7 and 97.8 to 99.7%, respectively.

Compared to Bacillus, 1654 out of 1800 spectra were correctly classified for all Clostridia, resulting in an overall accuracy of 91.9%. Some major misclassifications occurred between the closely related *C. butyricum*, *C. felsineum*, and *C. tertium*, with sensitivity values ranging from 80.7 to 90%, with *C. felsineum* having the lowest sensitivity of the three. The highest sensitivity values were reached by *C. perfringens* and *C. sporogenes* with 97.8 and 96.7%. In general, the sensitivity and specificity values ranged from 80.7 to 97.8 and 96.9 to 99.7% respectively. The classification on a species level for *P. sordellii* and *C. difficile* was not performed since only one representative of the species was used. The detailed classification results can be seen in Table 1 and Table S3A–D.

The finalized model was then challenged with a validation step consisting of independently cultivated bacterial strains utilized in the training data set. The validation data set consisted of the same seven *Bacillus*, five *Clostridium*, one *P. sordellii*, and 12 *C. difficile* strains. The word “independent” refers to the fact that the Raman spectra of these individually cultivated batches were not previously used in the model’s training. The previously built PCA-SVM model was used to predict those independent test data sets to check the sensitivity of the validation. These 25 validation samples were subjected to the same model of the respective genus, i.e., the Clostridia of the validation data set was tested against the Clostridia of the training set. The same procedure was performed for the *Bacillus* species as well. In Table S4A–D, 1157 of 1300 spectra were correctly validated on the cluster level, resulting in a sensitivity of 89%. Furthermore, this resulted in an overall genus validation accuracy of 89% as well, with accuracy rates for *Bacillus* at 60.8% and *Clostridium* at 58%. More detailed information on the validation results can be found in Table S4A–D.

The downstream SVM used to verify *Bacillus* and *Clostridium* species at the species level yielded results of varied degrees (Table S4A–D). Compared to the other genera, *Bacillus* validation had a 60.8% accuracy rate and many significant misclassifications. Since we tested the model by integrating aerobically and anaerobically cultivated *B. licheniformis* strains, the misclassifications with *B. atrophaeus*, as well as the poor sensitivity (71%), were not surprising. However, given that we also tested *B. cereus* under the same circumstances, it was intriguing to see that these isolates were misclassified as *B. mycoides* rather than *B. licheniformis*. These findings are likely because both bacteria belong to the *B. cereus* or *B. subtilis* groups, respectively. *B. spizizenii* (1/50) and *B. mycoides* (14/50) likewise had poor classification results and were frequently misclassified as *B. licheniformis* or *B. cereus*. One likely cause is that the model does not have sufficient validation spectra to appropriately categorize each species. For example, *B. spizizenii* had just 50 measured spectra for validation, but *B. licheniformis* or *B. cereus* had 200.

*Clostridium*, on the other hand, obtained a comparable validation accuracy of 58% despite various degrees of misclassification. Most significantly, *C. perfringens* was correctly identified to 100%, while the other species varied from 34 to 70%. Looking at the phylogenetic tree (Scheme 1), *C. butyricum*, *C. felsineum*, and *C. tertium* cluster relatively close together, which, along with the restricted number of spectra (50 each for validation), might lead to misclassification. *C. difficile* and *P. sordellii* were only verified at the genus level since only representatives of each were chosen. *P. sordellii* had several misclassifications with *C. difficile*, which might be attributed to their 71% genetic homology.^76^

Finally, an identification process was applied, in which the model was tested with one different bacterial strain of the same species per genus (Table S5A–D). On the cluster level, an overall accuracy of 95.8% (383/400) was achieved as well as 95.5% on the genus level. This conveys the robustness of the built PCA-SVM model since it suggests that it is indeed possible to identify unknown species of bacteria on a genus level with an established database. Unfortunately, the identification process on the species levels only achieved 78% for *Clostridium* and 14% for *Bacillus*. A plausible explanation for the misclassifications is that the to-be-identified species were different strains of *B. mycoides* and *B. thuringiensis*, which are closely related to *B. cereus* and belong to the *B. cereus* group.

It should also be mentioned that certain species’ identifications performed relatively well, while others failed. Here, the database would need to be expanded to include even more different species and strains, such as *P. sordellii*, which would aid in the identification process. The larger and more diverse the database and species, the more probable it is that identification will improve and become more accurate.

This study aimed to construct a thorough Raman data set of *Clostridium* and *Bacillus* as well as *C. difficile* and *P. sordellii*, which are frequently overlooked due to their phylogenetic relationship, cultivation conditions, and pathogenicity. Interestingly, Raman spectroscopy was effectively employed to distinguish Clostridia from former *Clostridium* species, *C. difficile* and *P. sordellii*. It also shows that compared to *Clostridium* species, *Bacillus* species are less complicated to measure and classify. Furthermore, it emphasizes the need to use the appropriate chemometric assessment and model type while investigating these microorganisms.

## Summary and Outlook

In light of the current limitations in medical diagnosis and Raman spectroscopy strategies pertaining to Clostridia, our objective was to present the first comprehensive study utilizing Raman spectroscopy and chemometric evaluation on the single bacterial cells to discriminate *Clostridium*, *Bacillus*, *Clostridioides*, and *Paraclostridium* on a genus and species level. It was also important to investigate whether the PCA-SVM model was able to successfully discriminate between the genera and species based on their phylogeny, cultivation conditions, and metabolic lifestyles.

The model was subjected to a challenge by being presented with 8775 collected spectra of single vegetative cells. The top-level classifier exhibited a discrimination power of 84.9% at the genus level, while the classification of the *Bacillus* and *Clostridium* species achieved accuracies of 94.7 and 91.9%, respectively. The inclusion of aerobic and anaerobic cultures of *B. cereus* and *B. licheniformis* were included in the training data set presented an additional challenge for the model, which was able to discriminate between the bacteria based on their species, rather than their cultivation conditions. Furthermore, the Raman spectra of aerobic and anaerobic cultivated bacteria demonstrated that aerobic respiration is dependent on cytochrome c, whereas aerobic respiration is not. Consequently, the model was able to differentiate between the genera and species based on their phylogenetic relationship, rather than on their cultivation conditions or metabolic activities. These promising results demonstrated that Raman spectroscopy combined with chemometrics is indeed a powerful and useful tool for the classification of *Clostridium* and *Bacillus* species, thus having great potential in clinical diagnosis.

The objective of this study was to create the first comprehensive Raman database on several environmentally and clinically relevant *Clostridium* species. The database was found to be stable and dependable to the extent that the estimated models were able to identify *Clostridium* from *Bacillus*, *Clostridioides*, and *Paraclostridium* at the genus level while also providing solid species classifications. Further research should focus on introducing additional *Clostridium* species and strains into the database to allow for strain-level differentiation, as well as strengthening the algorithms’ discriminating ability.

Once a database has been established, Raman spectroscopy can be a valuable alternative to the gold standard genetic analysis methods such as (real-time) PCR. It is a rapid approach that requires only an adapted isolation step to analyze samples without destroying them or requiring labels. It also needs less maintenance and is inexpensive when it comes to reagents and consumables. Its limitation lies on the level of sensitivity and specificity for detecting low abundances of bacteria or identifying particular genetic markers that PCR can provide.

One significant obstacle that Raman spectroscopy still has to address is the detection of polymicrobial infections. In general, a more complex sample will result in a more complicated database and the requirement for stronger discriminating algorithms. However, with ongoing advancements in data processing and the combination of Raman with other techniques, it could become a valuable tool in clinical diagnostics for polymicrobial infections.

## Supporting Information Available

Complete classification, validation, and identification confusion tables; detailed Raman band assignment for each Raman band (PDF)
