# Supplementary material for: Unveiling Microbial Diversity: Raman Spectroscopy’s Discrimination of Clostridium and Related Genera
Source: Anal Chem. 2024 Sep 18;96(39):15702–10. doi: 10.1021/acs.analchem.4c03280 (PMC11447666; doi:10.1021/acs.analchem.4c03280)
Supplement: Supplementary file 1 — ac4c03280_si_001.pdf [file ac4c03280_si_001.pdf]

Supporting Information

Unveiling Microbial Diversity: Raman Spectroscopy's Discrimination of *Clostridium* and Related Genera

Markus Salbreiter[1,3], Annette Wagenhaus[1,3], Petra Rösch[1,3,*] and Jürgen Popp[1,2,3]

1 Institute of Physical Chemistry, Friedrich Schiller University Jena, Helmholtzweg 4, D-07743 Jena, Germany

2 Leibniz Institute of Photonic Technology Jena - Member of the Research Alliance "Leibniz Health Technologies", Albert-Einstein-Str. 9, D-07745 Jena, Germany

3 InfectoGnostics Research Campus Jena, Philosophenweg 7, D-07743 Jena, Germany

Corresponding Author:

*E-Mail: petra.roesch@uni-jena.de. Phone: +49-36419-48381. Fax: +49-36419-48302.

TABLE OF CONTENT:

Table S1: *Clostridioides*, *Paraclostridium, Clostridium* and *Bacillus* species and strains used and their individual growth conditions.
Table S2: Assignment of the major Raman bands for the bacterial spectra according to literature.
Table S3A: Confusion table of the classification model of bacteria on a cluster level.
Table S3B: Confusion table of the classification model of bacteria on a genus level.
Table S3C: Confusion table of the classification of *Bacillus* species on a species level.
Table S3D: Confusion table of the classification of *Clostridium* species on a species level.
Table S4A: Confusion table of the validation of bacteria on a cluster level.
Table S4B: Confusion table of the validation of bacteria on a genus level.
Table S4C: Confusion table of the validation of *Bacillus* species on a species level.
Table S4D: Confusion table of the validation of *Clostridium* species on a species level.
Table S5A: Confusion table of the identification of bacteria on a cluster level.
Table S5B: Confusion table of the identification of bacteria on a genus level.
Table S5C: Confusion table of the identification of *Bacillus* species on a species level.
Table S5D: Confusion table of the identification of *Clostridium* species on a species level.
Table S6: Summary of the validation at the cluster, genus and species level.

<u>Table S1:</u> *Clostridioides*, *Paraclostridium*, *Clostridium* and *Bacillus* species and strains used and their individual growth conditions.

Species	Strain	Growth condition
Clostridioides difficile *(formerly Clostridium difficile)*	DSM27638, DSM27640, DSM28196, DSM28645, DSM27147, DSM27543, DSM27544, DSM27639, DSM1296, DSM12056, UK062, UK127,	Anaerobic
Paraclostridium sordellii *(formerly Clostridium sordellii)*	ATCC9714	Anaerobic
Clostridium sporogenes	DSM1664	Anaerobic
Clostridium felsineum	DSM53	Anaerobic
Clostridium tertium	UK059	Anaerobic
Clostridium perfringens	UK060, UK063, ATCC13124	Anaerobic
Clostridium butyricum	DSM2477, DSM2478	Anaerobic
Lysinibacillus sphaericus *(formerly Bacillus sphaericus)*	DSM28, DSM1867	Aerobic
Bacillus cereus	DSM345, DSM351	Aerobic and anaerobic
Bacillus licheniformis	DSM13, DSM15	Aerobic and anaerobic
Bacillus mycoides	DSM2048, DSM307	Aerobic
Bacillus spizizenii *(formerly Bacillus subtilis)*	DSM6399	Aerobic
Bacillus thuringiensis	DSM350, DSM2046	Aerobic
Bacillus atrophaeus	DSM675	Aerobic

DSM, Deutsche Sammlung von Mikroorganismen, Braunschweig, Germany; UK Universitätsklinikum Jena, Germany; ATCC, American Type Culture Collection, Manassas, Virginia, USA.

Table S2: Assignment of the major Raman bands for the bacterial spectra according to literature.

Bacillus Band position / cm^{-1}	*Clostridium* Band position / cm^{-1}	*Paraclostridium sordellii* Band position / cm^{-1}	*Clostridioides difficile* Band position / cm^{-1}	Band position according to reference / cm^{-1}	Assignment	Biomolecule	Reference
2936	2933	2930	2930	2909-2937	CH stretching vibration	Lipids	[1]
1658	1664	1664	1664	1650–1680	Amide I	Proteins	[2, 3]
1583				1583-1587	Cytochrome c	Proteins	[4]
1583	1574	1574	1574	1578	v_{ring}(C=C, C=N) of Guanine, Adenine	Nucleic acids	[2, 5]
1451	1448	1448	1448	1433-1468<br>1431-1481	CH_2/CH_3 deformation vibration of lipids and proteins	Lipids, Proteins	[6-8]
1397				1397	Cytochrome c	Proteins	[4]
	1337			1310-1348	CH-deformation vibration	Proteins, Lipids, Polysaccharides	[9]
1310				1311-1314	Cytochrome c	Proteins	[4]
1247	1256	1250	1250	1240-1265	Amide III	Proteins	[9]
1127				1130	Cytochrome c	Proteins	[10]
1001	1004	1004	1004	1004	Phenylalanine ring breathing vibration	Proteins	[8]
	851	851	851	854<br>850	C–C stretching vibration of proline, Out-of-plane ring deformation vibration of tyrosine	Proteins	[5, 9]
	824	824	824	824	Second ring breathing mode of tyrosine	Nucleic acids	[11]
779				780-786<br>786	Ring breathing modes of cytosine, uracil, and thymine, O—P—O stretching vibration DNA backbone	Nucleic acids	[5, 12]
749				749	Cytochrome c	Proteins	[4, 10]

<u>Tables S3:</u> A-D Confusion tables of the respective classification performances of the PCA-SVM model trained with three biological replicates (100 spectra / strain) of the bacteria of interest. Tables S3A and S3B show the classification results of the bacteria on a cluster and genus level, while the tables S3C and S3D show the classification results of *Bacillus* and *Clostridium* species.

<u>Table S3A:</u> Confusion table of the classification model of bacteria on a cluster level.

PREDICTION<br>TRUE	[1]	[2]	[3]	Sensitivity / %	Specificity / %	Accuracy / %
B [1]	3873	162	165	92.2	93.2	
C [2]	111	1272	417	70.7	90.8	82.5
CP [3]	200	482	2093	75.4	90.3	

<u>Table S3B:</u> Confusion table of the classification model of bacteria on a genus level.

PREDICTION<br>TRUE	[1]	[2]	[3]	[4]	Sensitivity / %	Specificity / %	Accuracy / %
Bacillus [1]	3881	136	169	14	92.4	94.0	
Clostridioides [2]	166	1978	314	11	80.1	91.8	
Clostridium [3]	106	369	1305	20	72.5	93.0	84.9
Paraclostridium [4]	1	9	6	290	94.8	99.5	

<u>Table S3C:</u> Confusion table of the classification of *Bacillus* species on a species level.

PREDICTION<br>TRUE	[1]	[2]	[3]	[4]	[5]	[6]	[7]	Sensitivity / %	Specificity / %	Accuracy / %
Bacillus atrophaeus [1]	285	9	1	1	1	3	0	95.0	99.0	
Bacillus cereus [2]	5	1119	4	42	4	0	26	93.2	93.2	
Bacillus licheniformis [3]	16	25	1139	1	5	14	0	94.9	99.4	
Bacillus mycoides [4]	6	16	0	278	0	0	0	92.7	98.9	94.7
Lysinibacillus sphaericus [5]	4	7	3	0	586	0	0	97.7	99.7	
Bacillus spizizenii [6]	5	4	8	0	0	282	1	94.0	99.5	
Bacillus thuringensis [7]	3	4	1	0	1	1	290	96.7	99.3	

<u>Table S3D:</u> Confusion table of the classification of *Clostridium* species on a species level.

PREDICTION<br>TRUE	[1]	[2]	[3]	[4]	[5]	Sensitivity / %	Specificity / %	Accuracy / %
Clostridium butyricum [1]	265	20	8	1	6	88.3	97.6	
Clostridium felsineum [2]	28	242	8	2	20	80.7	96.9	
Clostridium perfringens [3]	3	7	587	0	3	97.8	97.8	91.9
Clostridium sporogenes [4]	1	7	0	290	2	96.7	99.7	
Clostridium tertium [5]	4	13	11	2	270	90.0	97.9	

Tables S4: A-D Confusion tables of the respective validation performances of the PCA-SVM model applied to an independent data set of the bacteria of interest. Tables S4A and S4B show the validation results of the bacteria on a cluster and genus level, while the tables S4C and S4D show the validation results of *Bacillus* and *Clostridium* species.

Table S4A: Confusion table of the validation of bacteria on a cluster level.

PREDICTION<br>TRUE	[1]	[2]	[3]	Sensitivity / %	Specificity / %	Accuracy / %
B [1]	672	3	25	96.0	98.0	89.0
C [2]	1	197	52	78.8	94.9	
CP [3]	11	51	288	82.3	91.9	

Table S4B: Confusion table of the validation of bacteria on a genus level.

PREDICTION<br>TRUE	[1]	[2]	[3]	[4]	Sensitivity / %	Specificity / %	Accuracy / %
Bacillus [1]	674	19	7	0	96.3	98.2	89.0
Clostridioides [2]	8	258	34	0	86.0	91.7	
Clostridium [3]	0	44	204	2	81.6	95.5	
Paraclostridium [4]	3	20	6	21	42.0	99.8	

Table S4C: Confusion table of the validation of *Bacillus* species on a species level.

PREDICTION<br>TRUE	[1]	[2]	[3]	[4]	[5]	[6]	[7]	Sensitivity / %	Specificity / %	Accuracy / %
Bacillus atrophaeus [1]	47	1	2	0	0	0	0	94.0	91.4	**60.6**
Bacillus cereus [2]	1	122	1	71	0	1	4	61.0	86.2	
Bacillus licheniformis [3]	45	1	142	0	7	5	0	71.0	90.8	
Bacillus mycoides [4]	1	20	1	14	14	0	0	28.0	88.6	
Lysinibacillus sphaericus [5]	9	0	0	1	90	0	0	90.0	96.5	
Bacillus spizizenii [6]	0	5	42	2	0	1	0	2.0	99.1	
Bacillus thuringensis [7]	0	42	0	0	0	0	8	16.0	99.4	

Table S4D: Confusion table of the validation of *Clostridium* species on a species level.

PREDICTION<br>TRUE	[1]	[2]	[3]	[4]	[5]	Sensitivity / %	Specificity / %	Accuracy / %
Clostridium butyricum [1]	19	23	0	0	8	38.0	80.5	58.0
Clostridium felsineum [2]	26	24	0	0	0	48.0	73.5	
Clostridium perfringens [3]	0	0	50	0	0	100.0	100.0	
Clostridium sporogenes [4]	3	12	0	35	0	70.0	97.5	
Clostridium tertium [5]	10	18	0	5	17	34.0	96.0	

<u>Tables S5:</u> A-C Confusion tables of the respective identification performances of the PCA-SVM model applied to an independent data set of the bacteria of interest. Tables S5A and S5B show the identification results of the bacteria on a cluster and genus level, while the tables S5C and S5D show the identification results of *Bacillus* and *Clostridium* species.

<u>Table S5A:</u> Confusion table of the identification of bacteria on a cluster level.

PREDICTION<br>TRUE	[1]	[2]	[3]	Sensitivity<br>/ %	Specificity<br>/ %	Accuracy<br>/ %
B [1]	199	0	1	99.5	96.0	95.8
C [2]	7	91	2	91.0	98.0	
CP [3]	1	6	93	93.0	99.0	

<u>Table S5B:</u> Confusion table of the identification of bacteria on a genus level.

PREDICTION<br>TRUE	[1]	[2]	[3]	Sensitivity<br>/ %	Specificity<br>/ %	Accuracy<br>/ %
Bacillus [1]	200	0	0	100.0	92.5	95.5
Clostridioides [2]	1	96	3	96.0	100.0	
Clostridium [3]	14	6	86	86.0	99.0	

<u>Table S5C:</u> Confusion table of the identification of *Bacillus* species on a species level.

PREDICTION<br>TRUE	[1]	[2]	[3]	[4]	[5]	[6]	[7]	Sensitivity<br>/ %	Specificity<br>/ %	Accuracy<br>/ %
Bacillus atrophaeus [1]	0	0	0	0	0	0	0	n/a	100.0	14.0%
Bacillus cereus [2]	0	0	0	0	0	0	0	n/a	17.0	
Bacillus licheniformis [3]	0	0	0	0	0	0	0	n/a	99.5	
Bacillus mycoides [4]	0	97	1	0	0	0	2	0	97.0	
Lysinibacillus sphaericus [5]	0	0	0	0	0	0	0	n/a	100.0	
Bacillus spizizenii [6]	0	0	0	0	0	0	0	n/a	100:0	
Bacillus thuringensis [7]	0	69	0	3	0	0	28	28.0	98:0	

<u>Table S5D:</u> Confusion table of the identification of *Clostridium* species on a species level.

PREDICTION<br>TRUE	[1]	[2]	[3]	[4]	[5]	Sensitivity<br>/ %	Specificity<br>/ %	Accuracy<br>/ %
Clostridium butyricum [1]	0	0	0	0	0	n/a	81.0	78.0
Clostridium felsineum [2]	0	0	0	0	0	n/a	100.0	
Clostridium perfringens [3]	19	0	78	3	0	78.0	n/a	
Clostridium sporogenes [4]	0	0	0	0	0	n/a	97.0	
Clostridium tertium [5]	0	0	0	0	0	n/a	100.0	

<u>Table S6:</u> Summary of the validation at the cluster, genus and species level.

<table>
<tr><th colspan="2">Level</th><th>Number of spectra</th><th>Accuracy / %</th><th>Specificity / %</th><th>Sensitivity / %</th></tr>
<tr><td rowspan="3">Cluster</td><td>B</td><td>700</td><td rowspan="3">89.0</td><td>98.0</td><td>96.0</td></tr>
<tr><td>C</td><td>250</td><td>94.9</td><td>78.8</td></tr>
<tr><td>CP</td><td>350</td><td>91.9</td><td>82.3</td></tr>
<tr><td rowspan="4">Genus</td><td>Bacillus</td><td>700</td><td rowspan="4">89.0</td><td>98.2</td><td>96.3</td></tr>
<tr><td>Clostridium</td><td>250</td><td>91.7</td><td>86.0</td></tr>
<tr><td>Clostridioides</td><td>300</td><td>95.5</td><td>81.6</td></tr>
<tr><td>Paraclostridium</td><td>50</td><td>99.8</td><td>42.0</td></tr>
<tr><td rowspan="15">Species</td><td>Bacillus</td><td>700</td><td rowspan="8">60.6</td><td></td><td></td></tr>
<tr><td>Bacillus atrophaeus</td><td>50</td><td>91.4</td><td>94.0</td></tr>
<tr><td>Bacillus cereus</td><td>200</td><td>86.2</td><td>61.0</td></tr>
<tr><td>Bacillus licheniformis</td><td>200</td><td>90.8</td><td>71.0</td></tr>
<tr><td>Bacillus mycoides</td><td>50</td><td>88.6</td><td>28.0</td></tr>
<tr><td>Lysinibacillus sphaericus</td><td>100</td><td>96.5</td><td>90.0</td></tr>
<tr><td>Bacillus spizizenii</td><td>50</td><td>99.1</td><td>2.0</td></tr>
<tr><td>Bacillus thuringensis</td><td>50</td><td>99.4</td><td>16.0</td></tr>
<tr><td>Clostridium</td><td>250</td><td rowspan="7">58.0</td><td></td><td></td></tr>
<tr><td>Clostridium butyricum</td><td>50</td><td>80.5</td><td>38.0</td></tr>
<tr><td>Clostridium felsineum</td><td>50</td><td>73.5</td><td>48.0</td></tr>
<tr><td>Clostridium perfringens</td><td>50</td><td>100.0</td><td>100.0</td></tr>
<tr><td>Clostridium sporogenes</td><td>50</td><td>97.5</td><td>70.0</td></tr>
<tr><td>Clostridium tertium</td><td>50</td><td>96.0</td><td>34.0</td></tr>
</table>

References

(1) Czamara, K.; Majzner, K.; Pacia, M. Z.; Kochan, K.; Kaczor, A.; Baranska, M. Raman spectroscopy of lipids: a review. *Journal of Raman Spectroscopy* **2015**, *46* (1), 4-20.

(2) Maquelin, K.; Kirschner, C.; Choo-Smith, L. P.; van den Braak, N.; Endtz, H. P.; Naumann, D.; Puppels, G. J. Identification of medically relevant microorganisms by vibrational spectroscopy. *J. Microbiol. Meth.* **2002**, *51* (3), 255-271.

(3) Uzunbajakava, N.; Lenferink, A.; Kraan, Y.; Volokhina, E.; Vrensen, G.; Greve, J.; Otto, C. Nonresonant confocal Raman imaging of DNA and protein distribution in apoptotic cells. *Biophys. J.* **2003**, *84* (6), 3968-3981.

(4) Pätzold, R.; Keuntje, M.; Theophile, K.; Müller, J.; Mielcarek, E.; Ngezahayo, A.; Anders-von Ahlften, A. In situ mapping of nitrifiers and anammox bacteria in microbial aggregates by means of confocal resonance Raman microscopy. *J. Microbiol. Meth.* **2008**, *72* (3), 241-248.

(5) Huang, W. E.; Li, M.; Jarvis, R. M.; Goodacre, R.; Banwart, S. A. Shining light on the microbial world: the application of Raman microspectroscopy. *Adv. Appl. Microbiol.* **2010**, *70*, 153-186.

(6) Benevides, J. M.; Overman, S. A.; Thomas Jr, G. J. Raman, polarized Raman and ultraviolet resonance Raman spectroscopy of nucleic acids and their complexes. *J. Raman Spectrosc.* **2005**, *36* (4), 279-299.

(7) Managò, S.; Zito, G.; De Luca, A. C. Raman microscopy based sensing of leukemia cells: A review. *Opt. Laser Technol.* **2018**, *108*, 7-16.

(8) Töpfer, N.; Müller, M. M.; Dahms, M.; Ramoji, A.; Popp, J.; Slevogt, H.; Neugebauer, U. Raman spectroscopy reveals LPS-induced changes of biomolecular composition in monocytic THP-1 cells in a label-free manner. *Integrative Biol.* **2019**, *11* (3), 87-98.

(9) Azemtsop Matanfack, G.; Pistiki, A.; Rösch, P.; Popp, J. Raman Stable Isotope Labeling of Single Bacteria in Visible and Deep UV-Ranges. *Life* **2021**, *11*, 1003.

(10) Hu, S.; Morris, I. K.; Singh, J. P.; Smith, K. M.; Spiro, T. G. Complete assignment of cytochrome c resonance Raman spectra via enzymic reconstitution with isotopically labeled hemes. *J. Am. Chem. Soc.* **1993**, *115* (26), 12446-12458.

(11) Stöckel, S.; Schumacher, W.; Meisel, S.; Elschner, M.; Rösch, P.; Popp, J. Raman spectroscopy-compatible inactivation method for pathogenic endospores. *Appl. Environ. Microbiol.* **2010**, *76* (9), 2895-2907.

(12) Neugebauer, U.; Clement, J. H.; Bocklitz, T.; Krafft, C.; Popp, J. Identification and differentiation of single cells from peripheral blood by Raman spectroscopic imaging. *Journal of biophotonics* **2010**, *3* (8-9), 579-587.
